# Supplementary material for: Field Evaluation of Experimental Maize Hybrids for Resistance to the Fall Armyworm (Lepidoptera: Noctuidae) in a Warm Temperate Climate
Source: Insects. 2024 Apr 19;15(4):289. doi: 10.3390/insects15040289 (PMC11050381; doi:10.3390/insects15040289)
Supplement: Supplementary file 1 [file insects-15-00289-s001.zip › insects-2884122-supplementary.pdf]

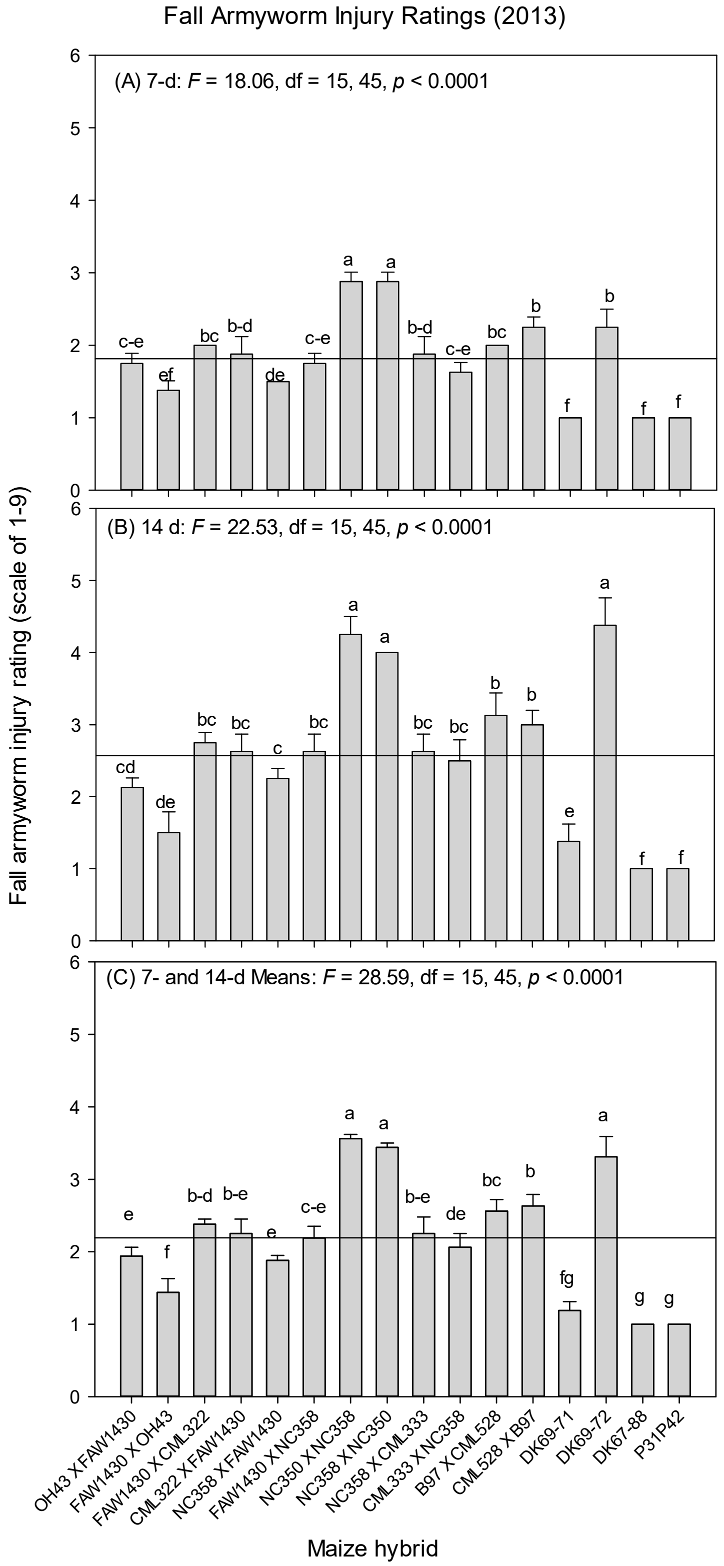

Figure S1. The *S. frugiperda* injury rating data from 2013 at 7-d (**A**) and 14-d (**B**) after the artificial infestation, and the means of the two ratings (**C**).

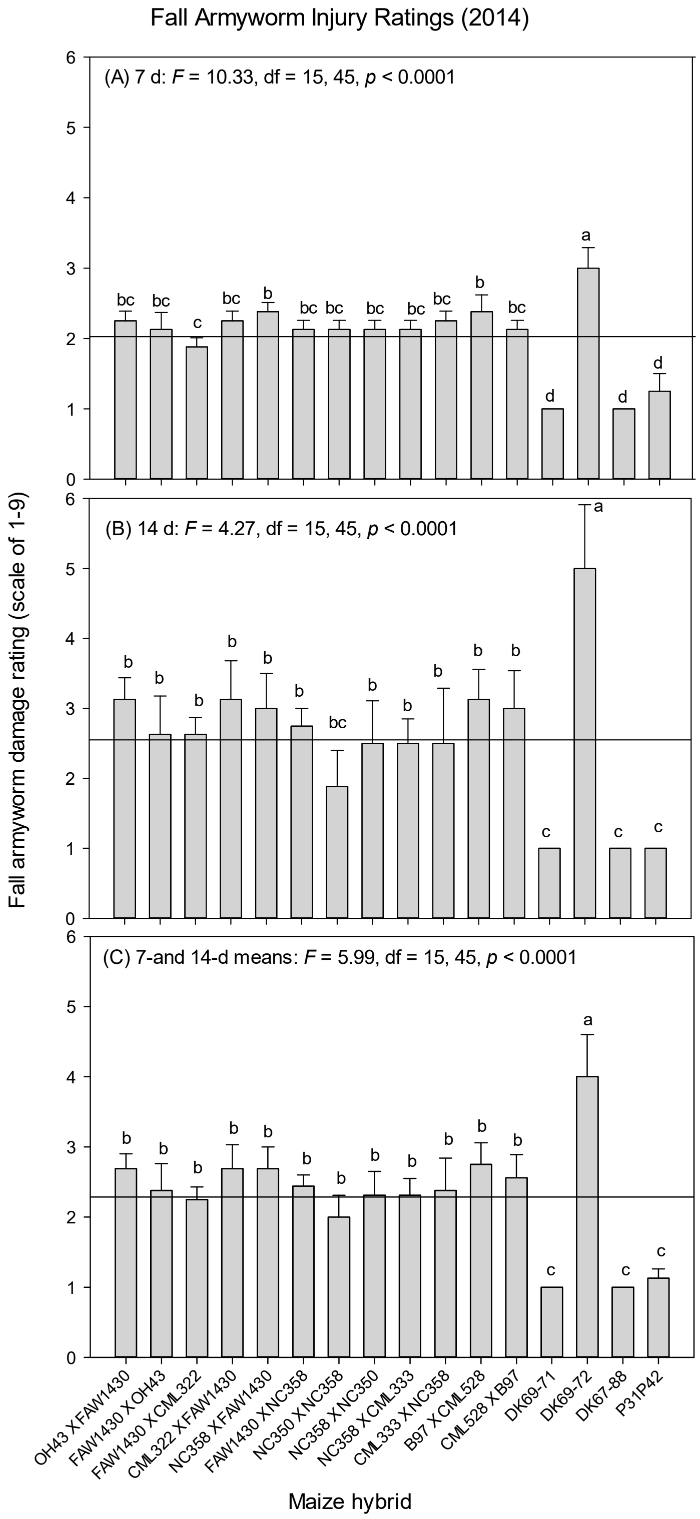

Figure S2. The *S. frugiperda* injury rating data from 2014 at 7-d (**A**) and 14-d (**B**) after the artificial infestation, and the means of the two ratings (**C**).

Table S1. Predator type and number from the 12 experimental hybrids and four commercial checks*.

Year	All predators	Hippo.	C.Mac.	C7	Harm.	Scy.	Hooded	Geo.	Orius	Nabid	Earwigs	Spiders
2013	**2.22 ± 0.18 a**	0.08±0.03	**0.14±0.04 a**	0.02±0.02	0.06±0.03	0.09±0.04	**0.08±0.03 a**	0.17±0.06	**1.36±0.13 a**	0.06±0.03	0.06±0.03	0.09±0.04
2014	**4.42 ± 0.31 b**	0.08±0.03	**0.36±0.08 b**	0.02±0.02	0.03±0.02	0.08±0.03	**0 b**	0.22±0.06	**3.42±0.25 b**	0.02±0.02	0.03±0.03	0.17±0.05

Year	Hybrids	All predators	Hippo.	C.Mac.	C7	Harm.	Scy.	Hooded	Geo.	Orius	Nabid	Earwigs	Spiders
2013	Oh43 x FAW1430	1.75 ± 0.49 abc	0	0 b	0	0	0.25±0.25	0 b	0	1.25±0.48 abc	0	0	0.25±0.25
	FAW1430 x Oh43	0.75±0.25 c	0	0 b	0	0	0	0 b	0	0.75±0.25 c	0	0	0
	FAW1430 x CML322	1.5±0.65 bc	0	0.25±0.25 ab	0	0	0	0.25±0.25 ab	0	1±0.71 bc	0	0	0
	CML322 x FAW1430	1.25±0.75 bc	0	0.25±0.25 ab	0	0	0	0 b	0.5±0.5	0.5±0.29 c	0	0	0
	NC358 x FAW1430	3±0.58 ab	0.25±0.25	0 b	0	0.5±0.29	0	0.25±0.25 ab	0.5±0.29	1.25±0.25 abc	0	0.25±0.25	0
	FAW1430 x NC358	2±0.41 abc	0	0 b	0.25±0.25	0	0	0 b	0.25±0.25	1.25±0.25 abc	0	0	0.25±0.25
	NC350 x NC358	3±0.91 ab	0	0 b	0	0	0	0.5±0.29 a	0.25±0.25	1.75±0.48 abc	0	0	0.5±0.29
	NC358 x NC350	1.75±0.48 abc	0	0 b	0	0	0	0.25±0.25 ab	0	1.5±0.29 abc	0	0	0
	NC358 x CML333	2.75±0.63 ab	0	0.5 b	0	0.25±0.25	0	0 b	0	1.25±0.25 abc	0	0.5±0.29	0.25±0.25
	CML333 x NC358	2.75±0.25 ab	0	0.25±0.25 ab	0	0	0	0 b	0	2.5±0.29 a	0	0	0
	B97x CML528	3±1.08 ab	0	0.5 b	0	0	0	0 b	0	2.25±0.85 ab	0	0.25±0.25	0
	CML528 x B97	2.25±0.63 abc	0.25±0.25	0.25±0.25 ab	0	0	0.25±0.25	0 b	0.5	1±0.41 bc	0	0	0
	DKC69-71	1.5±0.29 bc	0	0 b	0	0	0.25±0.25	0 b	0	1.25±0.48 abc	0	0	0
	DKC69-72	1.75±0.75 abc	0	0 b	0	0	0.25±0.25	0 b	0.5	0.75±0.25 c	0.25±0.25	0	0
	DKC67-88	3.5±0.87 a	0.25±0.25	0 b	0	0	0.25±0.25	0 b	0	2.25±0.25 ab	0.75±0.25	0	0
	P31P42	3±0.82 ab	0.5±0.29	0.25±0.25 ab	0	0.25±0.25	0.25±0.25	0 b	0.25±0.25	1.25±0.95 abc	0	0	0.25±0.25
2014	Oh43 x FAW1430	4.25±0.85 a	0	0.25±0.25 ab	0	0	0	0 b	1	2.75±0.48 a	0	0	0.25±0.25
	FAW1430 x Oh43	3.75±1.44 a	0	0 b	0	0.25±0.25	0	0 b	0	3.5±1.19 a	0	0	0
	FAW1430 x CML322	4.25±1.38 a	0	1±1 a	0	0	0	0 b	0.25±0.25	3±1.08 a	0	0	0

CML322 x FAW1430	5.75±0.85 a	0.25±0.25	1±0 a	0	0	0.25±0.25	0 b	0.25±0.25	3.75±0.48 a	0.25±0.25	0	0
NC358 x FAW1430	4±1.41 a	0.25±0.25	0 b	0	0	0	0 b	0.25±0.25	3.5±1.26 a	0	0	0
FAW1430 x NC358	3.75±0.85 a	0	0 b	0	0	0	0 b	0	3±1 a	0	0	0.75±0.48
NC350 x NC358	4.25±0.85 a	0	0.5±0.29 ab	0	0	0.25±0.25	0 b	0.25±0.25	3.25±0.75 a	0	0	0
NC358 x NC350	4.75±1.8 a	0	0 b	0	0	0.25±0.25	0 b	0	4.5±1.85 a	0	0	0
NC358 x CML333	4.25±1.03 a	0	0 b	0	0	0.25±0.25	0 b	0	3.75±1.11 a	0	0	0.25±0.25
CML333 x NC358	5±1.87 a	0.25±0.25	0.75±0.25 ab	0	0.25±0.25	0	0 b	0.25±0.25	3.5±1.26 a	0	0	0
B97 x CML528	5.75±1.25 a	0	0.75±0.48 ab	0	0	0	0 b	0.5	4±1.08 a	0	0	0.5±0.29
CML528 x B97	3±1.22 a	0.25±0.25	0 b	0	0	0	0 b	0	2.25±1.03 a	0	0.5±0.5	0
DKC69-71	5.5±1.55 a	0	0.25±0.25 ab	0	0	0	0 b	0.5	4.25±0.95 a	0	0	0.5±0.29
DKC69-72	4.5±0.87 a	0	0.25±0.25 ab	0	0	0.25±0.25	0 b	0.25±0.25	3.5±0.65 a	0	0	0.25±0.25
DKC67-88	5±2.35 a	0.25±0.25	0.25±0.25 ab	0.25±0.25	0	0	0 b	0	4±1.35 a	0	0	0.25±0.25
P31P42	3±0.91 a	0	0.75±0.25 ab	0	0	0	0 b	0	2.25±0.75 a	0	0	0

* For the pooled annual data (in the top two rows) $n = 64$, whereas $n = 4$ for the rest of rows in the table (or each hybrid entry within a year). The values in the same column with different letters were significantly different ($p < 0.05$).

Notes: All predators = total number of all predators recorded on an experimental plot at the seedling stage. The predator names are abbreviated as follows: Hippo. = the convergent lady beetle, *Hippodamia convergens* (Coleoptera: Coccinellidae); C.mac. = the pink-spotted lady beetle, *Coleomegilla maculata* (Coleoptera: Coccinellidae); C7 = the 7-spotted lady beetle, *Coccinella septempunctata* (Coleoptera: Coccinellidae); *Harm.* = the multicolored Asian lady beetle, *Harmonia axyridis* (Coleoptera: Coccinellidae); *Scy.* = the dusky lady beetle, *Scymnus* spp. (Coleoptera: Coccinellidae); Hooded = hooded (or flower) beetle, *Notoxus* spp. (Coleoptera: Anthicidae); Geo. = big-eyed bug, *Geocoris* spp. (Hemiptera: Geocoridae); *Orius* = the insidious flower bug (or minute pirate bug), *Orius insidiosus* (Hemiptera: Anthocoridae); Nabid = damsel bugs, *Nabis* spp. (Hemiptera: Nabidae); Earwigs = Dermapteran taxa identified as *Labidura riparia* (Labiduridae), and *Doru taeniatum* (Forficulidae); Spiders = all spider species observed; All predators = total number of all predators recorded on an experimental plot.

Table S2. The Pearson's Correlation Coefficients among the *S. frugiperda* injury ratings and predators recorded (n = 128).

	FAW7d	FAW14d	FAWm	Hippo.	C.Mac.	C7	Harm.	Scy.	Hooded	Geo.	Orius	Nabid	Earwigs	Spiders
**FAW14d**	**0.83**													
	**<0.0001**													
**FAWm**	**0.92**	**0.98**												
	**<0.0001**	**<0.0001**												
**Hippo.**	-0.10	-0.09	-0.10											
	0.24	0.32	0.27											
**C.Mac.**	0.00	0.01	0.01	0.08										
	0.97	0.93	0.94	0.37										
**C7**	-0.14	-0.11	-0.13	**0.20**	0.06									
	0.12	0.21	0.16	**0.03**	0.52									
**Harm.**	-0.09	-0.10	-0.10	0.07	-0.03	-0.03								
	0.30	0.24	0.24	0.41	0.70	0.75								
**Scy.**	-0.03	0.02	0.00	-0.09	-0.04	-0.04	0.06							
	0.76	0.82	0.96	0.32	0.67	0.67	0.47							
**Hooded**	0.13	**0.19**	**0.18**	-0.06	-0.09	-0.03	0.15	-0.06						
	0.15	**0.03**	**0.04**	0.51	0.30	0.78	0.10	0.49						
**Geo.**	0.03	0.03	0.03	0.00	0.06	-0.05	0.07	-0.01	0.00					
	0.74	0.74	0.73	0.97	0.53	0.54	0.45	0.92	0.98					
**Orius**	0.00	-0.11	-0.08	0.13	**0.21**	0.14	0.07	-0.08	-0.04	0.03				
	0.98	0.23	0.40	0.16	**0.02**	0.11	0.42	0.38	0.65	0.74				
**Nabid**	-0.17	-0.08	-0.11	0.09	-0.02	-0.03	-0.04	**0.23**	-0.04	0.09	-0.02			
	0.05	0.38	0.20	0.30	0.84	0.78	0.62	**0.01**	0.65	0.31	0.82			
**Earwigs**	0.03	0.10	0.08	**0.18**	0.03	-0.02	0.11	-0.06	-0.04	-0.01	-0.06	-0.04		
	0.77	0.27	0.39	**0.04**	0.74	0.79	0.22	0.51	0.67	0.89	0.53	0.67		
**Spiders**	0.09	0.05	0.06	-0.03	-0.05	**0.30**	0.02	-0.04	0.04	-0.02	0.07	-0.07	-0.07	
	0.34	0.61	0.49	0.77	0.58	**0.00**	0.82	0.69	0.67	0.86	0.41	0.41	0.43	
**All predators**	-0.01	-0.08	-0.06	**0.26**	**0.41**	**0.23**	**0.20**	0.04	0.02	**0.24**	**0.90**	0.10	0.07	**0.20**
	0.87	0.39	0.51	**0.00**	**<0.0001**	**0.01**	**0.03**	0.63	0.78	**0.01**	**<0.0001**	0.29	0.43	**0.02**

* In each table cell, values at the top row are the Pearson's coefficient (*r*) value, and the bottom is *p* value of the corresponding coefficient. The values in the bolded letters are significantly different ($p < 0.05$).

Notes: FAW7d and FAW14d are the FAW injury ratings at 7 and 14 d after the infestation, while the FAWm is the mean of 7 and 14 d within each year**;** the predator names are abbreviated as follows: Hippo. = the convergent lady beetle, *Hippodamia convergens* (Coleoptera: Coccinellidae); C.mac. = the pink-spotted lady beetle, *Coleomegilla maculata* (Coleoptera: Coccinellidae); C7 = the 7-spotted lady beetle, *Coccinella septempunctata* (Coleoptera: Coccinellidae); *Harm.* = the multicolored Asian lady beetle, *Harmonia axyridis* (Coleoptera: Coccinellidae); *Scy.* = the dusky lady beetle, *Scymnus* spp. (Coleoptera: Coccinellidae); Hooded = hooded (or flower) beetles, *Notoxus* spp. (Coleoptera: Anthicidae); Geo. = big-eyed bug, *Geocoris* spp. (Hemiptera: Geocoridae); *Orius* = the insidious flower bug (or minute pirate bug), *Orius insidiosus* (Hemiptera: Anthocoridae); Nabid = damsel bug, *Nabis* spp. (Hemiptera: Nabidae); Earwigs = Dermapteran insects identified as *Labidura riparia* (Labiduridae), and *Doru taeniatum* (Forficulidae); Spiders = all spiders observed without further identification; All predators = total number of all predators recorded on an experimental plot.

Table S3. Paired *t*-test results for *S. frugiperda* and predator survey data from 2013 and 2014 (*n* = 8).

Reciprocal pair*	Cross A vs. Cross B	Cross A	Cross B	Difference (Δ)*	Paired *t*-test
				Fall armyworm injury data	
1	Oh43 x FAW1430 vs. FAW1430 x Oh43	2.31 ± 0.18	1.91 ± 0.26	0.41 ± 0.28	*t* =- 1.45, df = 7, *p* = 0.19
2	FAW1430 x CML322 vs. CML322 x FAW1430	2.31 ± 0.09	2.47 ± 0.2	-0.16 ± 0.19	*t* = -0.8, df = 7, *p* = 0.45
3	NC358 x FAW1430 vs. FAW1430 x NC358	2.28 ± 0.21	2.31 ± 0.11	-0.03 ± 0.21	*t* = -0.15, df = 7, *p* = 0.89
4	NC350 x NC358 vs. NC358 x NC350	2.78 ± 0.33	2.88 ± 0.27	-0.09 ± 0.22	*t* = -0.43, df = 7, *p* = 0.68
5	NC358 x CML333 vs. CML333 x NC358	2.28 ± 0.15	2.22 ± 0.24	0.06 ± 0.33	*t* = 0.19, df = 7, *p* = 0.85
6	B97 x CML528 vs. CML528 x B97	2.66 ± 0.16	2.59 ± 0.17	0.06 ± 0.22	*t* = -0.28, df = 7, *p* = 0.78
7	Commercial control: Bt hybrid vs. non-Bt hybrid	1.09 ± 0.07	3.66 ± 0.33	-2.56 ± 0.34	***t* = -7.61, df = 7, *p* < 0.0001**
8	Commercial control: Bt hybrid vs. Bt hybrid	1 ± 0	1.06 ± 0.06	-0.06 ± 0.06	*t* = -1, df = 7, *p* = 0.35
				Predator profile data	
	Cross A vs. Cross B	Cross A	Cross B	Difference (Δ)*	**Paired *t*-test**
1	Oh43 x FAW1430 vs. FAW1430 x Oh43	3 ± 0.65	2.25 ± 0.88	0.75 ± 0.75	*t* = 1.0, df = 7, *p* = 0.35
2	FAW1430 x CML322 vs. CML322 x FAW1430	2.88 ± 0.88	3.5 ± 1	-0.63 ± 0.91	*t* = -0.69, df = 7, *p* = 0.51
3	NC358 x FAW1430 vs. FAW1430 x NC358	3.5 ± 0.73	2.88 ± 0.55	0.63 ± 1	*t* = 0.63, df = 7, *p* = 0.55
4	NC350 x NC358 vs. NC358 x NC350	3.63 ± 0.63	3.25 ± 1.03	0.38 ± 1.27	*t* = -0.3, df = 7, *p* = 0.78
5	NC358 x CML333 vs. CML333 x NC358	3.5 ± 0.63	3.88 ± 0.97	-0.38 ± 1.21	*t* = -0.31, df = 7, *p* = 0.77
6	B97 x CML528 vs. CML528 x B97	4.38 ± 0.92	2.63 ± 0.65	1.75 ± 0.9	*t* = 1.94, df = 7, *p* = 0.09
7	Commercial control: Bt hybrid vs. non-Bt hybrid	3.5 ± 1.05	3.13 ± 0.74	0.38 ± 0.92	*t* = 0.41, df = 7, *p* = 0.70
8	Commercial control: Bt hybrid vs. Bt hybrid	4.25 ± 1.19	3 ± 0.57	1.25 ± 1.35	*t* = 0.93, df = 7, *p* = 0.38

*The first six pairs are the true reciprocal crosses of the experimental hybrids, while the last two are not, they are the controls for the experiment, four commercial maize hybrids (see Table 1 for details). The values in the bolded letters are significantly different (*p* < 0.05).
